# Effect of School-Based Educational Intervention on Childhood Obesity in Croatian Urban and Rural Settings

**DOI:** 10.3390/children11070867

**Published:** 2024-07-17

**Authors:** Sara Cobal, Darija Vranešić Bender, Jasenka Gajdoš Kljusurić, Ivana Rumora Samarin, Željko Krznarić

**Affiliations:** 1 Croatian Medical Association, Šubićeva 9, 10 000 Zagreb, Croatia; zeljko.krznaric1@zg.ht.hr; 2 Unit of Clinical Nutrition, Division of Gastroenterology and Hepatology, Department of Internal Medicine, University Hospital Centre Zagreb, Kišpatićeva 12, 10 000 Zagreb, Croatia; dvranesi@kbc-zagreb.hr; 3 Faculty of Food Technology and Biotechnology, University of Zagreb, Pierottijeva 6, 10000 Zagreb, Croatia; jasenka.gajdos@pbf.unizg.hr (J.G.K.); ivana.rumora.samarin@pbf.unizg.hr (I.R.S.); 4 School of Medicine, University of Zagreb, Šalata 2, 10000 Zagreb, Croatia

**Keywords:** PETICA, children, obesity, nutrition, physical activity, habits

## Abstract

Background: Childhood obesity is a global public health concern. Development of effective public health interventions represents the only viable option for decreasing the prevalence of childhood obesity. The objective of this study was to assess the effectiveness of a school-based “PETICA—Play for Health” obesity prevention program in urban and rural areas in Croatia. Methods: This before-and-after study included 28 elementary schools in Croatia focused on pupils (*n* = 753, 2nd grade) and their guardians (*n* = 753) during the school year 2022/2023. “PETICA” multicomponent lessons and workshops (10) were implemented in the school curriculum and home settings (parents) as part of the intervention. Knowledge and lifestyle habit changes were evaluated via the questionnaire on knowledge and habits regarding nutrition and physical activity pre-/post-education. The Wilcoxon test for paired samples, Student’s t-test (dependent and independent samples), multiple Mann Whitney U tests (*p* < 0.05), Spearman’s correlation, and heatmaps were used. Results: The analysis showed a significant increase in acquired knowledge among pupils (both settings) and habits in the rural setting (increase in sports activities participation, breakfast, and vegetables consumption (*p* < 0.001, *p* = 0.003, *p* = 0.004)), decrease of sweets and salty snacks intake (*p* = 0.027, *p* = 0.011), and also, the link between parents’ and children’s physical activity levels (*p* = 0.028 vs. *p* = 0.022), emphasizing the importance of parents as healthy lifestyle role-models. Conclusion: The study shows that “PETICA” is an efficient school-based educational model that contributes to positive changes in children’s knowledge and lifestyle habits that are a prerequisite for childhood obesity prevention.

## 1. Introduction

Childhood obesity stands out as a significant public health concern in the 21st century [1,2], with far-reaching consequences spanning into adulthood. Its ramifications encompass a broad spectrum of health issues, including, but not limited to, obesity in later life, as well as endocrine, cardiovascular, and musculoskeletal disorders [2,3,4]. Moreover, it contributes to psychological challenges such as social stigma, depression, anxiety, and eating disorders in later years [5,6]. In addition, obesity can lead to poor quality of life as it often results in bullying at school, stigmatization, and poorer academic achievements [3,4]. Data on overweight and obesity in Europe have been studied extensively and vary according to sociodemographic characteristics such as gender, age, socioeconomic status, and rural/urban residence [5].

The studies conducted in Europe are inconclusive regarding the prevalence of obesity in urban and rural areas. They show a high prevalence of obesity in urban areas and, on the other hand, in some contexts in rural areas [7]. Croatian statistical data show that 31.2% of boys and 20.6% of girls at the age of 11 live with overweight and/or obesity [8]. Furthermore, when focusing on the region of residence in Croatia, the data show that the prevalence of obesity in children is higher in rural settings as opposed to urban ones [8].

In exploring the roots of overweight and obesity across diverse regions within a country, research suggests that the geographical setting of one’s residence and the level of urban development may serve as crucial determinants influencing dietary patterns and levels of physical activity (PA) [7].

Another important factor is the availability of school-based childhood obesity prevention programs, which have been developed with the aim of ensuring health across the lifespan [9] and that may reverse alarming health trends associated with obesity. They are a valuable public health tool that can improve the psychosocial factors associated with children’s knowledge, attitudes, and skills in relation to food and nutrition [9]. Studies have proposed that school-based childhood obesity prevention programs should focus on both nutrition education (NE) and PA aimed at changing lifestyle habits [10,11,12]. Due to the time spent in schools, they have been shown to be an efficient setting for the implementation of childhood obesity prevention programs [13]. In addition, literature reviews have shown that such programs should include both the child and the parents [14], involve teachers as educators [15,16], and should be implemented over a longer period of time (>6 months), contributing to knowledge acquisition and behavior change [13,17,18,19]. Lastly, they may have a more beneficial effect on body mass index (BMI) if implemented from an early age—6 to 12 years [17].

There have been scarce reports on childhood obesity prevention program development and implementation in Croatia. To the best of our knowledge, there are no comprehensive, extensive studies investigating the impact of childhood obesity prevention programs on the causes of overweight and obesity in different regions in Croatia.

The PETICA—play for health (PPH) childhood obesity prevention model is based on the Ensemble Prévenons l’Obésité Des Enfants (Fr. EPODE) methodology. The benefits of the EPODE methodology are the sustainability of the community-based programs developed worldwide, their plasticity, and the positive results achieved through continuous health-promotion activities focusing on nutrition and PA education [11].

At its core is the “Knowledge—Attitude—Behavior (KAB)” model which is based on the presumption that knowledge guides attitudes which lead to specific topic evaluation that ultimately influences behavior [20].

The aim of this study was to evaluate the impact of the PPH model on knowledge and lifestyle habits linked to the development of obesity in school-aged children and their parents, whilst considering potential differences in lifestyle characteristics of the studied population (in urban and rural areas) in Croatia.

## 2. Materials and Methods

### 2.1. Program Design

The PPH model is a multilevel Croatian school-based program, developed by an interdisciplinary team of experts, focused on children and their guardians but also on the local community. This model can be replicated and implemented in similar community settings. The science behind PETICA is based on nutritional guidelines for elementary school children, which emphasize the importance of a balanced diet, regular PA (at least 60 min per day or 12,000 steps) and limited sedentary behavior and screen time during the day (<1–2 h/day) [17]. Studies link certain behavioral risk factors to obesity, identifying them as crucial components in children’s diets. These factors include the consumption of energy-dense foods such as sweets, cakes, and pastries; highly processed snacks; sugar-sweetened beverages; and fast food. Additionally, skipping breakfast, low intake of fruits and vegetables, and decreased physical activity or inactivity are also significant contributors [17,21,22,23], which should be addressed through childhood obesity prevention initiatives. All of the above-mentioned factors are addressed through PPH educational materials focused on five (Cro. PETICA, the highest grade in school) topics. The key messages are explained and repeated in lessons and workshops for school-aged children—“eat breakfast”, “eat 5 portions of fruit and vegetables a day”, “drink 5 to 7 cups of water a day”, “sleep at least 9 to 11 h a night”, and “exercise at least 60 min a day”.

The PPH program is implemented through 10 lessons and workshops on the importance of healthy eating habits and PA activity, which are included in the school curriculum at 2nd-grade level. The children are taught by educators—teachers. Before program implementation, the teachers received a comprehensive 8 h training by experts (physicians, nutritionists, psychologists, and kinesiologists) and a step-by step manual explaining how to implement the PPH. All educational materials used as part of the PPH intervention are approved by the Croatian Education and Teacher Training Agency.

In addition, parents are educated during one school year using PPH materials based on the five age-appropriate topics mentioned above and participate in online workshops (5) on nutrition and PA conducted by nutritionists and kinesiologists.

The program ideally lasts for 3 consecutive years (3 cycles—2nd, 3rd and 4th grade).

### 2.2. Study Design

The study group consisted of school-age children and their parents (*n* = 753) from 28 elementary schools in Croatia (urban and rural settings) who participated in the PPH multicomponent educational model focused on childhood obesity prevention.

This before-and-after study was conducted during the school year 2022/2023 with the aim of determining the impact of the PPH program on (i) the knowledge and habits related to nutrition and PA of school-aged children in urban and rural settings; (ii) the differences and similarities perceived by parents for children and children per se in terms of healthy habits; (iii) the correlation between parental lifestyle behavior and children’s behavior depending on regional differences. Data were collected via questionnaires used to assess knowledge and habits (QKH) related to nutrition and PA habits. The study quantitatively and qualitatively observed data attained through the intervention and the statistical analysis comprised the Wilcoxon test for paired samples, Student’s t-test (dependent and independent samples), multiple Mann–Whitney U tests (*p* < 0.05) and Spearman’s correlation, and visual differences/similarities of results shown with heatmaps).

### 2.3. Intervention

The PPH program was implemented at 2nd-grade level as part of the school curriculum and all participants enrolled in 10 lessons and workshops (1 school hour each) regarding nutrition and PA as a prerequisite for healthy habit formation during the school year 2022/2023 in urban and rural regions in Croatia. The children were educated on animal- and plant-based foods, wholegrain foods, the Healthy Plate model, the Food Pyramid, and the importance of having a “rainbow on a plate”, in conjunction with the five key recommendations described in previous sections. The pupils had monthly lessons and workshops, which lasted up to 7 months cumulatively. Pupils’ knowledge and habits were examined via a questionnaire before and after education with PPH.

Parents were educated during the same school year with the help of PPH materials and online workshops. Parents’ knowledge, habits, and opinions regarding their children’s habits were assessed before and after PPH education using a questionnaire.

### 2.4. Participants and Recruitment

Participants from the urban region were from Zagreb County (the capital city of Croatia—Zagreb) and the participants from the rural region were from the least developed parts of three counties (Osječko-baranjska, Ličko-senjska, and Splitsko-dalmatinska) in Croatia. In order to obtain the most pronounced difference, areas with the highest and lowest Croatian development index were selected. The study was conducted in Croatian elementary schools (*n* = 28; urban region (18) and rural region (10)) that confirmed study participation after being invited to the study. The PPH program and study design were explained beforehand to the parents/guardians and only participants with written informed consent took part in the study. Those participants (children) who were not 8 years of age (2nd-grade pupils) or who were not guardians of the under-aged, did not have a signed written consent form, or did not participate in both rounds of evaluation were excluded from the study.

In order to achieve the required number of participants in the urban and rural regions, for which differences are expected according to the literature, the mean effect (effect size d = 0.5) using G*Power [24] was used to calculate the required number of participants in the mentioned groups. A priori power analysis (a = 0.05 and power (1-b error probability) = 0.95) was used to calculate the required sample sizes. Since the ratio between 2nd-grade pupils in rural and urban areas is 15% (according to the 2014–2016 development index), a value of 0.15 was taken as the allocation ratio (N2/N1). Accordingly, the total number of respondents required to ensure the desired level of power and minimization of erroneous conclusions was 460 (N1 = 402; N2 = 60). Given that the mentioned form of research was being conducted for the first time, and with the aim of preventing type II errors [25], 60% more respondents were targeted, resulting in a minimum number of 643 respondents in the urban area and 96 in the rural area.

The initial sample consisted of 1002 school-aged children and 908 parents. The final sample consisted of 753 pupils and their parents. The proportion of girls ranged from 49.5% in the urban region to 50.7% in the rural region, which is in accordance with the statistical data for Croatia. The data regarding education level, employment status, gender, and age of the parents are presented in Table 1.

### 2.5. Data Collection—Methods and Instruments

The method of data collection was questionnaires used to assess knowledge and habits related to nutrition and PA habits.

The questionnaire for pupils examined their general habits (5), dietary behavior (6), and knowledge on nutrition and physical activity (10).

The questionnaire for parents examined general data (4), general parent habits (4), knowledge (8), parents’ habits regarding dietary behavior (6), parents’ views on general habits of children (5), and parents’ views on their children’s dietary behavior (6). The questionnaire was designed based on a Likert scale of frequency. Participants were asked to rank questions assigning the answers based on a Likert scale with five gradations—“never”, “rarely”, “sometimes”, “often”, and “always”. For school-aged children, the answers were illustrated with a smiley face (from sad to happy) to ensure content comprehension.

**Figure 1:**
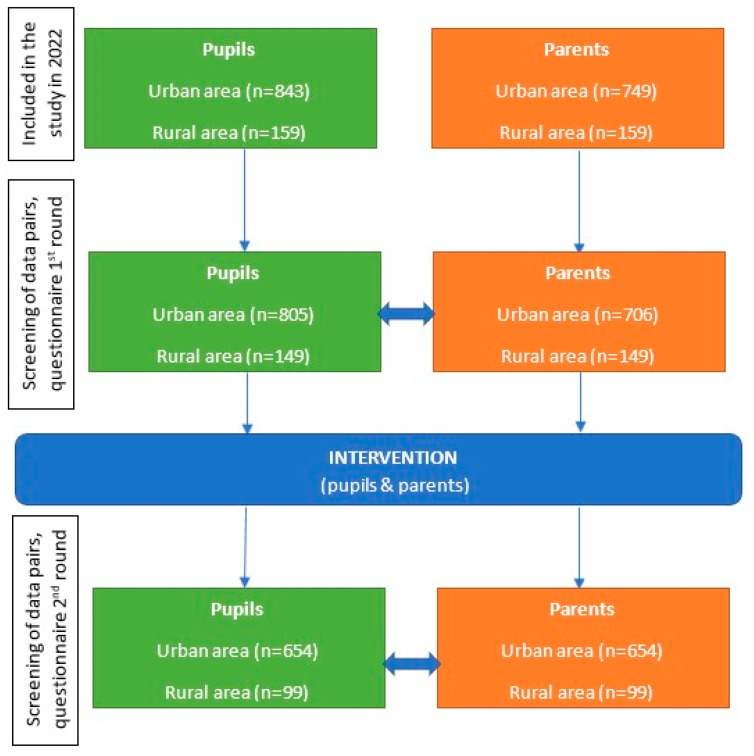
Flow chart of data collection to test similarities/differences (before and after the education) between the areas (urban and rural) for the observed group (pupils or parents), as well as between the groups in the same area.

The QKH was filled out independently before (first round) and after (second round) PPH education during the school year 2022/2023 in urban and rural areas. The pupils filled in the questionnaire during school lessons under the supervision of educators and their parents filled out the QKH in the home setting. The answers of a pupil and their parent made up a “pair”, which made it possible to analyze the differences and similarities between the observed groups (child vs. child (regionally) and parent vs. child). Also, in order to determine the effect of the “intervention”, it was necessary to compare the answers of the “pair” from the first round with the second round of the QKH. If either of the members of the “pair“ were missing in the second phase, the “pair” was deleted from the first round as well. Such sets of “pairs” enabled parent–child comparisons before and after education, which caused a decrease in the initial sample. Ultimately, 654 “pairs” were left in the urban region and 99 in the rural region. The flow chart of data collection is shown in Figure 1.

### 2.6. Data Analysis

Conducting surveys before (pre-testing—round 1) and after the education (post-testing—round 2) gave insights into changes related to dietary behavior and healthy habits among parents and children in urban and rural contexts. The data from the parents’ questionnaires were compared with the data collected from their children. When analyzing similarities and differences (parent/child; region 1 vs. region 2; round 1 vs. round 2) statistical tests such as the Wilcoxon test for paired samples, the t-test for dependent samples, and multiple Mann–Whitney U tests were used. The significance level applied was 5%. Spearman’s correlation was used to examine the degree of association of the variables recorded in the form of an ordinal variable modality. Heatmaps were also used as a qualitative display of similarities and differences (which are identified by colors), which was used to determine similarities and/or differences in the physical activity level of the subjects.

All the data were compiled in databases and statistically analyzed using the statistical programs XLSTAT (version 2014, Addinsoft, Paris, France) and IBM SPSS Statistic for Windows, released 2021 (version 28, Armonk, NY, USA, IBM Corp), following the guidelines for statistical methods [26].

### 2.7. Ethics

The study was conducted in accordance with the Helsinki Declaration, Croatian Health Care Law (NN 100/18, 125/19, 147/20, 119/22, 156/22, 33/23, 36/24) [27], and the protocol of the study was reviewed and approved with written consent by the Croatian Medical Association Ethical Committee (1950/T).

## 3. Results

The results of the study showed similarities and differences between urban and rural pupils depending on whether they were tested before or after the education. Also, we observed differences between parents and children in urban and rural areas depending on pre- or post-education testing. The study also provided valuable insights and it is an addition to the existing research on the importance of parents as healthy-lifestyle role models that children tend to emulate. The results regarding parents’ knowledge and habits are described elsewhere.

### 3.1. Pupils’ Knowledge and Habits—Urban vs. Rural Region

The analysis showed a statistically significant change in knowledge about nutrition and PA among pupils from both urban and rural settings. Pupils from the urban areas acquired more knowledge regarding vegetable and water intake, had a better understanding of the Food Pyramid and Healthy Plate model, knowledge regarding plant-based foods, and the amount of recommended PA. On the other hand, the pupils from rural areas acquired knowledge regarding the importance of fruit and water intake and the Healthy Plate model. The results of pupils’ knowledge related to nutrition and PA are shown in Table 2.

The importance of these results lies in the fact that knowledge about healthy dietary behavior is a prerequisite for the formation of healthy habits, which are associated with a non-communicable disease risk reduction [20,28]. In the urban region, there were significant changes in almost 70% of responses (for 7 out of 10 questions), while the remaining frequency remained unchanged: it is necessary to (i) have breakfast every day (95.9 vs. 98.2%) and (ii) consume at least two pieces of fruit a day (91.4 vs. 91.1%), but also to sleep at least 8 h (83% before and after the education). In the rural areas, after the education, statistically significant differences were found in the responses on daily fruit consumption (92.9% vs. 98% after the education) and water consumption. After the education, pupils showed an increase in acquired knowledge regarding the recommended daily water intake (27.3% increased to 50.5%). The education also clarified doubts about the composition of the Healthy Plate and 47.5% of them disagreed that it consisted exclusively of fruits, vegetables, and grains, which represents a significant increase (*p* = 0.003) compared to the initial 33.3% of pupils who agreed with this statement.

The results of the statistical analysis regarding general and dietary habits of pupils in different regions (Table 3) showed that no statistically significant difference was found for any of the pupils’ responses from the urban areas, while amongst pupils in the rural areas, the time spent watching TV decreased significantly (*p* = 0.006) from the initial 47.5% of those who (“sometimes”, “often”, and “always”) watched TV more than 2 h per day to 32.3% after the education. An extremely positive effect of education can be seen in the change in habits related to their activity, such as the increase in time spent playing outdoors (*p* = 0.011) and participation in sports activities (*p* < 0.001). A particular improvement in eating habits can be seen in the decrease in the proportion of pupils who “never” or “rarely” eat breakfast (from 13.1% to 8.1%) and in the increase in the proportion of those who eat breakfast regularly (from 48.5% to 67.7% (*p* = 0.003)). Pupils from the rural region also increased their intake of vegetables (*p* = 0.004) and decreased the intake of sweets (*p* = 0.027) and salty snacks (*p* = 0.011).

The differences in pupils’ answers regarding their knowledge and habits, depending on the region (urban vs. rural), and before (round 1) and after (round 2) the conducted education, are summarized and shown in Table S1. Only three statements did not change after the education and showed no significant differences in the pupil’s responses in the observed regions, namely, (i) the need for daily water consumption; (ii) the need for regular breakfast consumption; and (iii) the habit of consuming sweets every day.

### 3.2. Parents and Children—Habits

The analysis of general data on parents (Table 1) showed that the average age of parents was higher in the urban areas compared to the rural areas (41.2 vs. 36.0 years) and the sample showed that most respondents in both areas were female (78.3% vs. 91.9%). The majority of parents from the urban area had a college/master’s degree (44.8%), whereas parents from the rural area predominantly had high school diplomas (74.7%). Lastly, parents from the urban area had a higher rate of full-time employment (88.7% vs. 40.4%).

The pairing of parents and children represents a unique set of results provided by this study and it gave insight into the children’s perceptions of habits (Table 3) and the parents’ perceptions of their children’s habits (Table 4) in the urban and rural regions.

**Figure 2:**
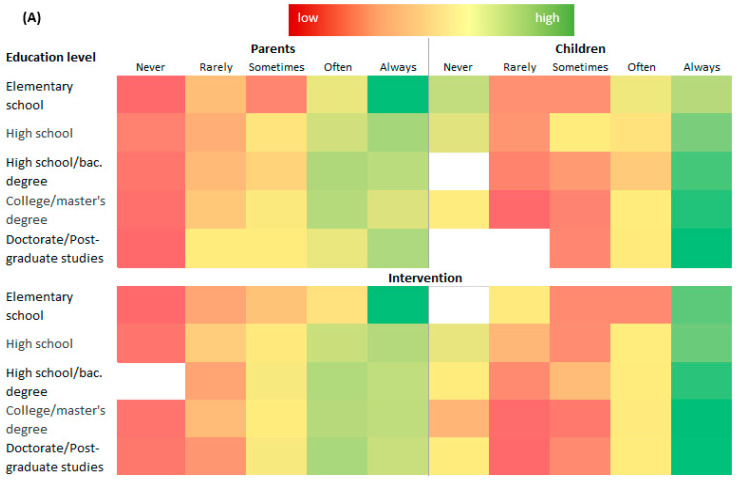

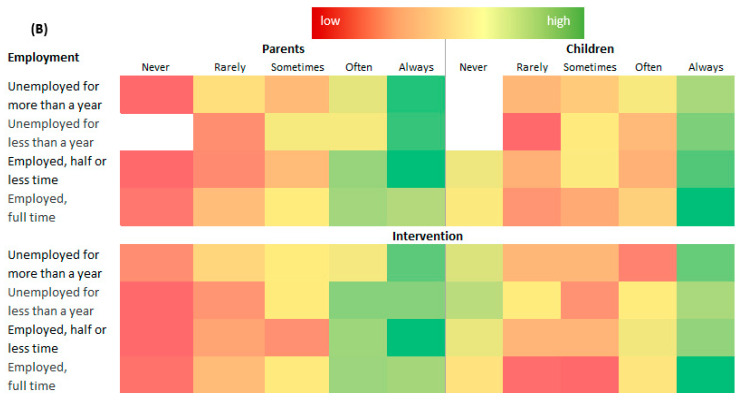
Heatmaps relating parents’ personal physical activity habits and children’s sport activity based on the parents’ level of education (**A**) and the employment status (**B**) before and after the educational intervention.

The results of the statistical analysis of parents’ perceptions of their child’s eating habits in different regions showed that there was no statistically significant difference found in urban areas, while parents’ perceptions in rural areas showed a significant decrease in time spent watching TV (*p* = 0.0320), from 45.5% of those who “never” or “rarely” watch TV for more than 2 h a day to 60.7% after the education. A positive effect of the education was seen in the children’s expected change in habits related to the increase in time spent playing outdoors (*p* = 0.0006). In addition, the parents confirmed a particular improvement in their children’s eating habits due to reduced intake of daily snacks (*p* = 0.0139). The data showed that the perceptions of children in rural areas were somewhat consistent with those of their parents. This is a valuable finding due to the challenges that researchers are faced with when investigating the impact of childhood obesity prevention models that include children as study participants. However, to account for additional factors such as the level of education and/or parental employment, it is necessary to utilize the tools of multivariate analysis. Heatmaps are used in multivariate analysis of qualitative data [29] and such presentation made it possible to link parents’ habits and their relationship with children’s habits (regular physical activity) while considering factors such as their employment status and level of education. The study thus found evidence that parents’ behavior is linked to that of their children (Figure 2).

In the presented heatmap, the colors visually connect the segments of the responses within the parent-related category (education level or employment status), which are arranged in rows, with the corresponding responses of the children (Figure 2), both before and after educational interventions. The pre-test showed a significant relationship between parents’ PA habits and children’s participation in sports activities (n1 = n2 = 753) before the intervention and after the application of bivariate correlations (Spearman’s rho: r = 0.061, *p* = 0.027). This significance remained (r = 0.065, *p* = 0.021) after the intervention/education of both children and parents, as determined by the post-test (Wilcoxon matched pairs test conducted on original input data for the stated PAs). The heatmaps indicate significant changes in the PA levels before and after the education when the educational level of parents is considered (Figure 2A). The data for children in both urban and rural areas, regardless of the education level, showed a dominance in the “often” and “always” category for the statement “I go to a sport at least twice a week” (Table 3). Unfortunately, when employment status is considered, it becomes clear that there is a correlation between the additional financial support required and participation in additional extracurricular activities (Figure 2B). Thanks to the features of the heatmaps (Figure 2), which indicate similarities and differences with their colors, there is an obvious shift towards more frequent PA for both groups (parents and children), which is just an additional confirmation of the positive impact of the education on the importance of regular PA. The dominance of yellow and green colors compared to red and orange is evident in the observed line for a certain group of respondents. These valuable data add to the literature emphasizing the importance of nutrition and PA education as a key driver for healthy habit formation in families.

## 4. Discussion

The development of effective interventions focused on childhood obesity prevention programs is not a straightforward process. The results of a systematic review by Belich et al. [30] evaluated interventions for the prevention of childhood obesity, including a total of 56 randomized controlled trials, quasi-experimental studies, and natural experiments. The majority of obesity prevention interventions analyzed focused on the school setting, with fewer interventions focusing on preschool, home settings, or the community level. The results showed more consistent and positive outcomes for school-based interventions that consisted of multiple components, combining diet, PA segments, and a home-based element, compared to the same type of intervention that differed in the combination of components and settings [30]. In addition, a systematic review and meta-analysis [31] confirmed that multicomponent school-based interventions contribute to BMI reduction. However, the authors stressed that further research is needed regarding the prevalence of included components in the intervention and differences between countries [30]. A review conducted by Lambrinou et al. [32] showed that elementary schools seem to be vital settings for nutrition and PA education implementation due to time spent in schools and the supportive environment. Also, the authors emphasized effective strategies for childhood obesity prevention: the importance of teacher involvement in the intervention as educators, having been previously trained by health professionals; and parent involvement through educational materials that encourage changes and promote healthy-lifestyle behaviors in the home environment [32]. Nga et al. [33] conducted a systematic review that showed positive results of nutrition education on pupils’ food preferences, nutrition knowledge, improved physical activities, enhanced BMI z-scores, and waist circumference. Hui Ho, Cheng, and Lua [31] examined the impact of school-based interventions on childhood obesity by quantitatively observing twelve cluster-RCTs. The results of the study found that school-based interventions resulted in positive behavioral changes of reduced junk food intake and increased intake of fruit and vegetables. In addition, a review [34] showed that models based on both nutrition and PA components have favorable effects on multiple health outcomes such as anthropometric parameters and also eating behaviors.

The results of the PPH intervention in the observed groups, in urban and rural settings of Croatia, are in line with the obesity prevention strategies and results of the previously mentioned studies. The results of the study also confirm the effectiveness of the Croatian PPH model and its contributions to a significant increase in acquired knowledge related to nutrition and PA among school-aged children in the urban and rural settings. To the best of our knowledge, this is the first study addressing the topic of healthy habit formation in school-aged children and their guardians in various regional contexts in Croatia through an educational childhood obesity prevention program such as PPH.

It is important to educate children about healthy dietary patterns and PA activity from an early age in order to enhance their nutrition and PA literacy, as studies show that this correlates with improved nutrition knowledge, dietary and PA behavior, sedentary behavior, screen time, and weight status [35,36]. In addition, according to the KAB model, the prerequisite for behavior change is the accumulation of knowledge [37], which was addressed and achieved through the PPH program. However, the study showed no statistically significant changes in the habits of school-aged children in the urban areas. Pirgon et al. [38] reported that a rapid increase in the level of urbanization leads to significant shifts in dietary behaviors and PA levels, which consequently increase the risk of obesity in children. Urban sprawl has also been pointed out as an obesogenic environmental component that may influence the formation of healthy habits in children living in those settings [39] as urban planning may not consider public health and the risk of obesity, e.g., buildings that are not suitable for walking in their surroundings [38]. On the other hand, Crouch et al. [40] argued that children from rural areas may have more opportunities to be physically active outdoors which could explain why habit formation was seen in the observed rural region group, but not in the urban one. Another plausible explanation for the change in habits only in the rural setting is the fact that the education was performed in smaller groups of pupils, considered a preferable teaching style, that might have impacted the level of acquired knowledge, the motivation, and attitudes of the children, but also of the educators [41]. In the study by Randell, Udo, and Warne [42], teachers were found to influence attitudes regarding PA and play a key role in pupil participation in outdoor activities. Lastly, a systematic review by Klein et al. [43] emphasized that nutrition interventions can contribute to reducing obesity by extension, even if they do not achieve statistically significant outcomes in all domains.

Another important point addressed by this study is the parental role in promoting healthy eating and PA behaviors in the home environment, shaping them and their significant impact on obesity prevention [44,45,46,47,48,49,50,51]. Furthermore, the research results complement the findings of recent literature on parental involvement and education through childhood obesity prevention programs, as their influence may extend into the larger community and additionally contribute to the reduction of the obesity epidemic [2,47]. A plausible explanation for the observed discrepancy between acquired knowledge and habit formation in children from the urban region compared to the rural region is the high employment rates of parents in the urban region (88.7%) observed in this study. A review of the recent literature showed that a lack of parental presence and routines within a family can significantly impact overall family health by disrupting the home environment and affecting the ability to promote health [45]. This lack of structure and support can, in turn, hinder the effective transfer of healthy behaviors to children [45].

### 4.1. Limitations

Despite the valuable insights provided by this study, there are limitations that need to be acknowledged. Firstly, no anthropometric parameters were considered and linked to the collected data. The focus of this study was on evaluating the effectiveness of PPH on healthy habit formation that, in turn and by extension, contributes to the decreased risk of overweight and obesity. Secondly, this study has a limitation pertaining to the age group of participants, as the questionnaire was administered to school-aged children (8-year-olds). A question has been raised regarding the accuracy of self-assessment in children of this age, particularly in assessing their habits. This could have a corresponding effect on the correlations found between the variables. To mitigate bias, efforts were made to enhance the reliability of the responses. The questionnaires were self-administered in the school setting under the supervision of educators, and habits were also evaluated from the guardian perspective, reducing the risk of misleading results.

Lastly, another noteworthy limitation lies in the diverse backgrounds and motivations of the educators responsible for intervention implementation. Although all educators underwent the same training, the different backgrounds and motivation levels could lead to biases that affect the consistency of intervention implementation and compliance among both educators and pupils. Awareness of these limitations is important for the interpretation of the study’s findings.

### 4.2. Implications for Future Research and Practice

Changes in the prevalence of childhood obesity through public health interventions have shown modest results so far [52]. Schools are important settings for pediatric obesity prevention through promotion of adequate dietary behavior and PA habits [52]. Further research is needed to investigate the effectiveness of school-based childhood obesity prevention programs on non-communicable disease development in diverse regional settings. Variations in socio-cultural contexts, environmental factors, type of components included, and to what extent they may influence the success of such interventions in urban and rural contexts, influencing habit formation of parents, as role models, and children should further be investigated. Tailoring strategies to the unique characteristics and needs of various regions can enhance the efficacy of public health initiatives. In addition, there is a need for studies to identify the optimal age for the implementation of public health protocols to maximize efficiency. Pinpointing the developmental stage at which interventions yield the most significant impact can guide the design of targeted strategies for health promotion. Moreover, it is crucial to extend research beyond immediate outcomes and track the long-term effects of interventions through later life.

In summary, future research should focus on identifying the key components that obesity prevention programs should comprise to maximize outcomes, assessing regional differences that may impact the effectiveness of interventions, understanding the key factors influencing healthy habit formation, and tracking long-term outcomes. These research niches will contribute significantly to the development of evidence-based public health strategies with broader applicability and impact.

## 5. Conclusions

This study shows that “PETICA—Play for Health” is an efficient Croatian educational public health model that contributes to positive changes in school-aged children’s knowledge and lifestyle habits that are a prerequisite for childhood obesity prevention. The results also confirm the importance of parents as healthy lifestyle role-models that should clearly be a part of multilevel, multicomponent public health interventions.

In summary, the results provide valuable insights for designing comprehensive health initiatives focused on childhood obesity prevention contributing to healthy growth and wellbeing that can extend to adult age and healthy communities.

## Figures and Tables

**Table 1:** Baseline data about parents of pupils (participants of the PETICA program) (*n* = 753) from two regions—urban and rural.

	Urban (*n* = 654)	Rural (*n* = 99)
Education level (% in the observed region)		
Elementary school	1.2	16.2
High school	30.1	74.7
Bachelor’s degree	15.9	5.1
College/master’s degree	44.8	4.0
Doctorate/postgraduate studies	8.0	0.0
Employment status (% in the observed region)		
Unemployed for more than a year	5.2	39.4
Unemployed for less than a year	1.1	11.1
Retired	0.2	1.0
Employed, half- or less time	4.9	8.1
Employed, full-time	88.7	40.4
Gender of the respondents (% in the observed region)		
Male	21.7	8.1
Female	78.3	91.9
Age of the respondents (years)		
All (average [range])	39.5 [23–55]	35.1 [24–46]
Male (average [range])	41.1 [26–55]	39.0 [28–46]
Female (average [range])	39.0 [23–52]	34.7 [24–46]

Note: Values are presented as percentage in the observed region (%), mean, or range (ge of the parents).

**Table 2:** Pupils’ knowledge related to healthy eating habits in different regions (*n* = 753).

Questions	Round 1		Round 2		*p*-Value
	Yes	No	Yes	No	
Urban (*n* = 654)					
I should eat breakfast every day.	95.9	2.6	98.2	1.5	0.481
I should eat fruit at least twice per day.	91.4	6.9	91.1	8.7	0.483
I need to eat vegetables only once per day.	23.7	74.5	10.1	89.4	0.001 **
I should drink no more than 4 glasses of water per day.	56.0	42.4	29.7	70.0	0.000 **
Fruit is at the top of the Food Pyramid.	33.5	64.2	15.6	84.1	0.000 **
I need to sleep less than 8 h per night.	15.3	83.0	16.4	83.0	0.784
A Healthy Plate should contain only fruits, vegetables, and grains.	50.8	46.8	35.9	63.5	0.001 **
Foods of plant origin are: cereals, fruit and milk.	35.2	61.3	28.0	71.4	0.077 *
A Healthy Plate does not contain fat.	51.8	46.2	35.2	64.2	0.000 **
I need to be active only for half an hour every day.	34.1	64.1	20.2	79.5	0.002 **
Rural (*n* = 99)					
I should eat breakfast every day.	93.9	5.1	97.0	3.0	0.341
I should eat fruit at least twice per day.	92.9	7.1	98.0	2.0	0.049 *
I need to eat vegetables only once per day.	21.2	75.8	25.3	72.7	0.345
I should drink no more than 4 glasses of water per day.	70.7	27.3	48.5	50.5	0.000 **
Fruit is at the top of the Food Pyramid.	39.4	58.6	32.3	64.6	0.169
I need to sleep less than 8 h per night.	26.3	71.7	27.3	69.7	0.757
A Healthy Plate should contain only fruits, vegetables, and grains.	65.7	33.3	51.5	47.5	0.003 **
Foods of plant origin are: cereals, fruit and milk.	50.5	46.5	47.5	51.5	0.393
A Healthy Plate does not contain fat.	40.4	57.6	43.4	53.5	0.475
I need to be active only for half an hour every day.	36.4	62.6	36.4	62.6	1.000

* Correlation is significant at the 0.05 level; ** correlation is significant at the 0.01 level. The Wilcoxon test for paired samples was used to determine statistically significant differences in knowledge, which was monitored in two rounds via a survey questionnaire (round 1 vs. round 2). The analysis was performed at a significance level of 5%.

**Table 3:** General and dietary habits of pupils in different regions (*n* = 753).

Questions	Round 1					Round 2					*p*-Value
	Never	Rarely	Sometimes	Often	Always	Never	Rarely	Sometimes	Often	Always	
Urban (*n* = 654)											
I watch television for a long time every day (more than 2 h).	31.3	34.7	24.5	5.5	2.8	35.5	35.5	22.0	4.0	2.1	0.850
I play games on a computer or a console every day (more than 2 h).	53.1	18.8	15.0	8.0	4.0	50.3	21.4	15.6	8.0	3.2	0.955
I play outdoors for at least 1 h every day.	2.0	7.8	16.7	31.8	40.8	1.5	5.0	13.1	26.6	52.1	0.214
I go to a sport activity at least twice a week (swimming, soccer, dance, martial arts, tennis…).	12.8	3.4	5.4	7.5	70.5	10.1	3.2	3.8	9.8	71.6	0.781
I sleep a lot every night.	2.6	6.0	11.2	22.8	56.7	2.3	5.8	9.3	26.1	55.8	0.931
I have breakfast every morning.	1.1	3.2	7.0	9.2	77.5	2.1	4.4	6.0	13.0	73.9	0.483
I drink water every day (more than 5 glasses per day).	2.8	6.4	13.1	20.3	55.2	1.2	2.6	10.9	19.9	64.7	0.271
I eat vegetables every day (at least 3 times per day).	8.1	18.2	26.9	24.9	19.9	5.7	11.6	26.0	26.9	29.1	0.110
I eat fruit every day (at least 2 times per day).	5.2	6.3	13.1	25.2	47.9	2.3	5.7	11.5	22.6	56.0	0.472
I eat sweets every day (candies, biscuits, cakes…).	12.7	25.7	27.2	19.4	12.4	11.0	35.5	28.6	14.7	8.6	0.174
I eat snacks every day (chips, sticks…).	15.7	39.4	27.5	10.2	5.4	19.9	41.0	22.9	9.2	6.1	0.712
Rural (*n* = 99)											
I watch television for a long time every day (more than 2 h).	19.2	33.3	38.4	8.1	1.0	32.3	33.3	24.2	7.1	1.0	0.006 **
I play games on a computer or a console every day (more than 2 h).	33.3	20.2	27.3	8.1	9.1	42.4	23.2	17.2	9.1	6.1	0.099
I play outdoors for at least 1 h every day.	0.0	6.1	16.2	23.2	52.5	1.0	0.0	5.1	20.2	68.7	0.011 *
I go to a sport activity at least twice a week (swimming, soccer, dance, martial arts, tennis…).	34.3	3.0	5.1	11.1	43.4	28.3	11.1	4.0	13.1	42.4	0.000 **
I sleep a lot every night.	3.0	2.0	7.1	21.2	66.7	2.0	3.0	10.1	21.2	59.6	0.576
I have breakfast every morning.	2.0	11.1	19.2	19.2	48.5	1.0	7.1	9.1	15.2	67.7	0.003 **
I drink water every day (more than 5 glasses per day).	3.0	4.0	12.1	26.3	53.5	0.0	3.0	15.2	22.2	59.6	0.253
I eat vegetables every day (at least 3 times per day).	9.1	12.1	31.3	34.3	13.1	3.0	18.2	23.2	32.3	22.2	0.004 **
I eat fruit every day (at least 2 times per day).	3.0	7.1	18.2	31.3	38.4	0.0	10.1	16.2	33.3	38.4	0.321
I eat sweets every day (candies, biscuits, cakes…).	14.1	32.3	26.3	17.2	10.1	12.1	32.3	38.4	12.1	4.0	0.027 *
I eat snacks every day (chips, sticks…).	10.1	31.3	36.4	11.1	11.1	10.1	38.4	27.3	19.2	5.1	0.011 *

* Correlation is significant at the 0.05 level; ** correlation is significant at the 0.01 level. Note: Values are presented as percentage (%). To determine a statistically significant difference in the answers to the same question (for two rounds), a *t*-test for dependent samples was used at a significance level of 5%.

**Table 4:** Parents’ perceptions related to their child’s eating habits (*n* = 753).

Questions	Round 1					Round 2					*p*-Value
	Never	Rarely	Sometimes	Often	Always	Never	Rarely	Sometimes	Often	Always	
Urban (*n* = 654)											
My child watches television for more than 2 h per day.	19.1	38.4	31.0	7.3	0.8	19.4	40.8	31.0	7.0	0.9	0.9951
My child plays on the computer and/or console games for more than 2 h per day.	39.1	28.7	20.9	7.2	0.5	37.5	32.0	22.9	6.4	0.6	0.9448
My child plays outdoors every day for at least 1 h per day.	0.3	4.1	21.6	50.8	19.3	0.2	2.6	15.7	50.3	30.4	0.0696
My child is involved in an organized type of exercise at least twice a week (swimming, soccer, dance, martial arts, tennis…).	6.3	4.1	6.4	10.1	69.4	5.4	4.0	6.1	9.8	73.5	0.9817
My child sleeps ≥ 9 h per night.	0.3	2.8	6.4	30.3	56.7	0.2	1.5	4.7	34.4	58.4	0.7955
My child eats breakfast every morning.	0.3	1.1	3.2	16.1	75.8	0.2	1.7	3.8	14.7	78.6	0.9437
My child drinks 5 or more glasses per day.	0.3	4.3	12.8	39.8	39.1	0.0	2.8	11.3	41.4	43.7	0.8011
My child eats 3 or more servings of vegetables every day.	5.8	19.9	38.4	25.8	6.1	5.2	15.6	40.4	30.6	7.3	0.6984
My child eats 2 or more servings of fruit every day.	1.8	8.6	26.9	40.2	18.5	2.0	8.0	28.3	42.2	18.3	0.9941
My child eats desserts and/or sweets (candies, biscuits, cakes…) every day.	3.1	18.7	32.3	32.9	9.5	2.6	22.8	31.3	33.5	8.7	0.8972
My child eats snacks (chips, sticks) every day.	9.3	45.0	32.6	8.0	1.5	9.2	48.3	30.1	10.4	1.1	0.8583
Rural (*n* = 99)											
My child watches television for more than 2 h per day.	10.1	35.4	44.4	7.1	2.0	15.2	45.5	30.3	6.1	3.0	0.0320 *
My child plays on the computer and/or console games for more than 2 h per day.	22.2	34.3	21.2	16.2	2.0	31.3	28.3	26.3	11.1	2.0	0.1087
My child plays outdoors every day for at least 1 h per day.	0.0	3.0	10.1	49.5	36.4	0.0	0.0	2.0	39.4	56.6	0.0006 **
My child is involved in an organized type of exercise at least twice a week (swimming, soccer, dance, martial arts, tennis…).	22.2	10.1	15.2	17.2	33.3	0.0	8.1	21.2	12.1	37.4	0.2364
My child sleeps ≥ 9 h per night.	0.0	3.0	8.1	25.3	62.6	0.0	3.0	7.1	27.3	59.6	0.9796
My child eats breakfast every morning.	0.0	3.0	5.1	22.2	68.7	0.0	4.0	8.1	21.2	66.7	0.6880
My child drinks 5 or more glasses per day.	0.0	4.0	12.1	35.4	46.5	0.0	3.0	14.1	30.3	51.5	0.7615
My child eats 3 or more servings of vegetables every day.	4.0	21.2	40.4	24.2	6.1	4.0	27.3	38.4	24.2	5.1	0.7356
My child eats 2 or more servings of fruit every day.	1.0	12.1	29.3	42.4	14.1	4.0	11.1	29.3	40.4	15.2	0.0531
My child eats desserts and/or sweets (candies, biscuits, cakes…) every day.	1.0	14.1	41.4	32.3	8.1	2.0	19.2	41.4	25.3	11.1	0.2400
My child eats snacks (chips, sticks) every day.	3.0	18.2	47.5	25.3	5.1	3.0	30.3	34.3	25.3	7.1	0.0139 *

* Correlation is significant at the 0.05 level; ** correlation is significant at the 0.01 level. Note: Values are presented as percentage (%). To determine a statistically significant difference in the answers to the same question (for two rounds), a *t*-test for dependent samples was used at a significance level of 5%.

## Data Availability

The data presented in this study are available on request from the corresponding author due to restrictions of privacy.

## Supplementary Materials

The following supporting information can be downloaded at: https://www.mdpi.com/article/10.3390/children11070867/s1, Table S1: Differences in pupils’ responses regarding their knowledge and habits, depending on the region and the conducted round of data collection (*n* = 753).
