# Supplementary material for: Effect of School-Based Educational Intervention on Childhood Obesity in Croatian Urban and Rural Settings
Source: Children (Basel). 2024 Jul 17;11(7):867. doi: 10.3390/children11070867 (PMC11276343; doi:10.3390/children11070867)
Supplement: Supplementary file 1 [file children-11-00867-s001.zip › children-3088470-supplementary.pdf]

Table S1. Differences in Pupils' responses regarding their Knowledge and Habits, depending on the Region and the Conducted Round of Data Collection (n_{total} = 753)

Questions	***p* - value Urban vs Rural**	
	Round 1	Round 2
I watch television for a long time every day (more than 2 hours).	0.0023**	0.5139
I play games on a computer or a console every day (more than 2 hours).	0.0005**	0.5193
I play outdoors for at least 1 hour every day.	0.1790	0.0007**
I go to a sport activity at least twice a week (swimming, soccer, dance, martial arts, tennis...).	0.0000**	0.0000**
I sleep a lot every night.	0.0195*	0.4013
I have breakfast every morning.	0.0000**	0.3803
I drink water every day (more than 5 glasses per day).	0.5737	0.7432
I eat vegetables every day (at least 3 times per day).	0.0436*	0.0924
I eat fruit every day (at least 2 times per day).	0.1613	0.0051**
I eat sweets every day (candies, biscuits, cakes...).	0.6706	0.0747
I eat snacks every day (chips, sticks…).	0.0331*	0.0033**
I should eat breakfast every day.	0.2675	0.3838
I should eat fruit at least twice per day.	0.8646	0.0000**
I need to eat vegetables only once per day.	0.5753	0.0003**
I should drink no more than 4 glasses of water per day.	0.0007**	0.0001**
Fruit is at the top of the Food Pyramid.	0.2321	0.0001**
I need to sleep less than 8hours per night.	0.0116*	0.0085**
A Healthy Plate should contain only fruits, vegetables and grains.	0.0030**	0.0015**
Foods of plant origin are: cereals, fruit and milk.	0.0022*	0.0001**
A Healthy Plate does not contain fat.	0.0191*	0.0542
I need to be active only for half an hour every day.	0.6763	0.0006**

* Correlation is significant at the 0.05 level; ** Correlation is significant at the 0.01 level.

Note: Values are presented as percentage (%). To determine a statistically significant difference in the answers to the same question (for two rounds), a t-test for independent samples was used at a significance level of 5%.
